# Molecular assessment of three species of *Anilocra* (Isopoda, Cymothoidae) ectoparasites from Caribbean coral reef fishes, with the description of *Anilocra brillae* sp. n.

**DOI:** 10.3897/zookeys.663.11415

**Published:** 2017-03-27

**Authors:** Rachel L. Welicky, Kerry A. Hadfield, Paul C. Sikkel, Nico J. Smit

**Affiliations:** 1 Water Research Group, Unit for Environmental Sciences and Management, Potchefstroom Campus, North-West University, Private Bag X6001, Potchefstroom, 2520, South Africa; 2 Department of Biological Sciences, Arkansas State University, P.O. Box 599, State University, AR, 72467, USA

**Keywords:** *Anilocra haemuli*, *Anilocra chromis*, brown chromis, Caribbean, coral reef, Cymothoidae, fish ectoparasite, French grunt, Isopoda, molecular analysis, new species, parasite, red hind, taxonomy

## Abstract

A morphological review and molecular characterization of *Anilocra haemuli* Bunkley Williams & Williams, 1981, were completed using specimens collected from *Haemulon flavolineatum* Desmarest, 1823 (French grunt) and *Epinephelus guttatus* Linnaeus, 1758 (red hind). Molecular and morphological data suggest that the isopods parasitizing *H. flavolineatum* and *E. guttatus* are different species. The specimens collected from *E. guttatus* are recognized as a new species, *Anilocra brillae* **sp. n.** Differences between *Anilocra brillae* **sp. n.** and *A. haemuli* include but are not limited to the pleonites 1–3 of *A. brillae* **sp. n**. being wider than 4–5 and 4–5 subequal, whereas the pleonites 1–2 of *A. haemuli* are wider than 3–5, and 3–5 are subequal. The seventh pereopod of *A. brillae* **sp. n.** is proportionally larger, has more robust setae, and the setae are distributed more extensively over the articles when compared to *A. haemuli*. Additionally, this study provides the first genetic characterization of three *Anilocra* spp. from the Caribbean, and is based on mitochondrial cytochrome c oxidase subunit gene (COI) for *A. haemuli* from *H. flavolineatum*, *A. brillae* **sp. n.** from *E. guttatus*, and *A. chromis* Bunkley Williams & Williams, 1981 from *Chromis multilineata* Guichenot, 1853.

## Introduction

In the past half-century, taxonomic studies on the fish parasitic isopod genus *Anilocra* Leach, 1818, have reported nine species from the Caribbean (Bunkley Williams and Williams 1981) and 12 species from Australia (Bruce 1987). This genus of parasite parasitizes the external surfaces of marine fish hosts that inhabit subtropical, tropical, and temperate waters (Smit et al. 2014). Host specificity of species of *Anilocra* is highly variable, such that different Caribbean *Anilocra* have been identified as family, genus, and species specific (i.e. Bunkley Williams and Williams 1981, Bruce 1987). For example, *Anilocra holocentri* Bunkley Williams & Williams, 1981 has been reported only to infest *Holocentrus adscensionis* Osbeck, 1765, whereas *Anilocra chaetodontis* Bunkley Williams & Williams, 1981 has been reported to infest four members of the genus *Chaetodon* Linnaeus, 1758. *Anilocra haemuli* Bunkley Williams & Williams, 1981 is the only Caribbean species reported to infest fishes from two families: Haemulidae and Serranidae. Anecdotal accounts from both parasitologists and ecologists suggest that records of *A. haemuli* from Haemulids and Serranids may in fact be two species given the differences in the biology and ecology of these host fishes.

To evaluate this claim a review of *Anilocra haemuli* morphology using specimens from both the Haemulidae and Serranidae families is warranted. The original description of *A. haemuli* was published before molecular approaches were used to aid in confirming the morphological classification of organisms. In the original description, careful attention was taken to describe *A. haemuli* as type specimens were collected from the same host and locality (Bunkley Williams and Willams 1981). Nevertheless, multiple morphologically similar species of *Anilocra* may have been identified as *A. haemuli* because there was no other method to verify if these specimens represented multiple species.

An increasing number of ecological studies are using *Anilocra* to understand trophic level dynamics (Roche et al. 2013, Binning et al. 2014), and *A. haemuli* infestation has been associated with altering *H. flavolineatum* behavior and condition (Welicky and Sikkel 2014, 2015, Welicky et al. in press). To facilitate future ecological and evolutionary studies on *Anilocra*–host interactions, the identity of *Anilocra haemuli* is here validated using both a morphological redescription and a molecular analysis.

## Materials and Methods

### Specimen collection

In August 2016, *Epinephelis guttatus* Linnaeus, 1758, (family Serranidae) (n = 8) parasitized by a cymothoid isopod of the genus *Anilocra* were collected by free-divers using a modified cast net (Sikkel et al. 2004, 2006, Welicky et al. 2013) from Guana Island, British Virgin Islands (BVI). The *Anilocra* specimens were removed from host fish using forceps and then stored in 80% ethanol. *Anilocra haemuli* from *H. flavolineatum* Desmarest, 1823, (family Haemulidae) (St. John, USVI, n = 4, 2011; n = 2, 2012; n = 1, 2013; Guana Island, BVI, n = 1, 2012; n = 2, 2013; St. Thomas, USVI, n = 2) were collected in a similar manner as part of other studies, and initially frozen and then preserved in 80% ethanol. To include a third and more morphologically distinct *Anilocra* sp., *Anilocra chromis* Bunkley Williams & Williams, 1981, infesting *Chromis multilineata* Guichenot, 1853 (St. John USVI, n = 8, 2012-2013) were also collected. These were collected in a similar manner to those of *A. haemuli* from *H. flavolineatum*.

### Molecular analysis

Of the specimens collected, genomic DNA was extracted from eight *Anilocra* from *E. guttatus*, seven *A. haemuli* from *H. flavolineatum*, and eight *A. chromis* from *C. multilineata* using a rapid DNA extraction method as described in the KAPA Express Extract Kit (Kapa Biosystems, Cape Town, South Africa). Polymerase chain reactions (PCR) were used to amplify a 710 basepair fragment of the mitochondrial cytochrome c oxidase subunit gene (COI) using the primer sets LCO 1490 and HCO 2198 (Folmer et al. 1994). PCR was performed using 12.5 μl Thermo Scientific DreamTaq PCR master mix (2×) (2× DreamTaq buffer, 0.4 mM of each dNTP, and 4 mM MgCl2), 1.25 μl of each primer, 1 μl DNA, and 9 μl of PCR-grade nuclease free water (Thermo Scientific, Vilnius, Lithuania). Total volume per reaction was 25 μl, and PCR reactions were conducted using a ProFlex™ PCR thermal cycler (Applied Biosystems by Life Technologies). Reactions were amplified under the following PCR conditions: Stage 1, 94°C for 5min, Stage 2, 36 cycles of 94°C for 30s, 47°C for 50s, 72°C for 2min, and Stage 3, 72°C for 10min. PCR products were sent to a commercial sequencing company (Inqaba Biotechnical Industries (Pty) Ltd, Pretoria, South Africa) for purification and sequencing in both directions. Obtained sequences were assembled, and chromatogram-based contigs were generated using Geneious Ver. 9.1. Sequences were aligned and trimmed to the length of the shortest sequence using MEGA 7 bioinformatics software program (http://www.megasoftware.net)

Using BLASTn (Basic Local Alignment Search Tool; http://www.ncbi.nlm.nih.gov/blast), the obtained sequences were verified as belonging to the Isopoda. Pair-wise distance (p-distance) using the Kimura 2-parameter model and nucleotide differences were determined in MEGA7. Supplemental comparisons among the sequences of this study and those available for *Anilocra* sp. from GenBank were also determined. Newly-generated sequences for *Anilocra* spp. were deposited in GenBank under the accession numbers: *A. haemuli*: KY562752, KY562753, KY562754, KY562755, KY562756, KY562757, KY562758; *A. brillae* sp. n.: KY562744, KY562745, KY562746, KY562747, KY562748, KY562749, KY562750, KY562751; *A. chromis*: KY562736, KY562737, KY562738, KY562739, KY562740, KY562741, KY562742, KY562743.

### Morphological data

*Anilocra haemuli* from *Haemulon flavolineatum* and *Anilocra* from *Epinephelus guttatus* were examined using material previously collected by Ernest Williams and Lucy Bunkley-Williams during 1976–1977 and 1983 and reported in Bunkley-Williams and Williams (1981). Additionally, specimens from each host were collected using the aforementioned methods as part of other studies conducted in the US Virgin Islands (USVI) and British Virgin Islands (BVI) during 2011–2016. Isopods were processed according to the techniques described in Hadfield et al. (2010, 2013). Descriptions were prepared using DELTA (Descriptive Language for Taxonomy, Coleman et al. 2010) using a general character set for the Cymothoidae (Hadfield et al. 2014, 2016). Ratios and measurements were rounded off to one decimal place and were made using maximum values of the specific measured article. Ratios and measurements were taken from the female (♀) and transitional stage (TS) specimens used for the drawings and presented herein as figures. Pleotelson length (TL) and width (W) for all specimens examined are reported. All measurements are reported in milliimeters (mm). Classification follows Brandt and Poore (2003).

## Results

### Molecular analyses

Comparative sequence analysis indicated that there were three distinct species present in the samples based on the host species, *A. haemuli* from *H. flavolineatum, A. chromis* from *C. multilineata* and another undescribed species of *Anilocra* from *E. guttatus*. The intraspecific divergence observed within species was as follows: *A. haemuli*, 1–6 nt (0.6%); *A.* sp. n., 1–4 nt (0.3%); and *A. chromis*, 1–6 nt (0.7%) (Suppl. materials 1 and 2). The interspecific divergence between pairs of *Anilocra* spp. was as follows: *A. haemuli* and *A.* sp. n., 12–19 nt (4%); *A. haemuli* and *A. chromis*, 31–37 nt (9%); and *A. chromis* and *A.* sp. n., 31–37 nt (8%) (Suppl. materials 1 and 2). The interspecific divergence ranged between 104–109nt (30%) for all of our specimens combined and those available on GenBank (Suppl. materials 1 and 2).

### Taxonomy

#### Genus Anilocra

Taxon classificationAnimaliaIsopodaCymothoidae

Leach, 1818

Anilocra Leach 1818: 348, 350. Desmarest 1825: 306; Edwards 1840: 255; Dana 1853: 747; Schioedte and Meinert 1881: 100; Gerstaecker 1882: 231; Richardson 1905: 25; Hale 1926: 210; Schultz 1969: 153; Kensley 1978: 78; Kussakin 1979: 281; Brusca 1981: 140; Brusca and Iverson 1985: 45. Bruce 1987: 89; Trilles 1975: 303; Trilles 1994: 55; Thatcher and Blumenfeldt 2001: 270.
Canolira Leach, 1818: 350.
Epichthyes Herklots, 1870: 122.

##### Diagnosis.

A detailed diagnosis was given by Bruce (1987).

##### Type species.

The type species for this genus is *Anilocra cuvieri* Leach, 1818, junior synonym of *Anilocra physodes* (Linnaeus, 1758) (see Bruce 1987); by subsequent designation (Kussakin 1979).

Leach (1818) described three species: *Anilocra cuvieri*, *Anilocra mediterranea* Leach, 1818, and *Anilocra capensis* Leach, 1818 without designating a type species. *A. cuvieri* was designated as the type species by Kussakin (1979). Both *Anilocra cuvieri* and *A. mediterranea* were synonymized with *A. physodes* (Trilles 1975; Ellis 1981).

##### Remarks.

The body of female *Anilocra* is dorsally symmetrical and strongly vaulted. The posterior margins of their cephalon are smooth and straight, and the rostrum is more blunt than pointed. The rostrum folds into the area between the antennula bases. The antennula is shorter than the antenna. The posterolateral margins of the pereonites are not produced. Coxae 1–3 are short, posteriorly rounded and do not form a rounded point posteriorly, whereas coxae 4–6 are longer, less rounded and more elongate than coxae 1–3, and form a rounded point posteriorly. The pereopods gradually increase in size towards the posterior.

In the Cymothoidae, the external-attaching genera include but are not limited to *Anilocra*, *Nerocila* Leach, 1818, *Renocila* Miers, 1880, *Creniola* Bruce, 1987, and *Pleopodias* Richardson, 1910. *Anilocra* can be distinguished from *Nerocila* by the posterior margin of the cephalon, which is conspicuously trilobed in *Nerocila*, whereas the posterior margin of the cephalon of *Anilocra* is not tri-lobed to weakly tri-lobed. The posterolateral pereonite margins of *Nerocila* are more produced, elongate and pointed than that of *Anilocra*. In the Caribbean, some species of *Anilocra* and *Renocila* share numerous similarities, but in *Anilocra* pereopod 6 is shorter in length than pereopod 7, whereas in *Renocila* pereopods 6 and 7 are of similar length. To date the genera *Creniola* and *Pleopodias* have not been reported from the Caribbean.

#### Anilocra haemuli

Taxon classificationAnimaliaIsopodaCymothoidae

Bunkley Williams & Williams, 1981

Figs 1 , 2 , 3 , 4

Anilocra haemuli (Part) Bunkley Williams and Williams 1981: 1004–1014, figs 4–5; Williams and Williams 1985: 92–95; Bunkley-Williams and Williams 1998: 862–869; Bunkley-Williams et al. 1999: 311-314; Bunkley-Williams et al. 2006: 175–188; Welicky and Sikkel 2014: 1018–1026, 2015: 1437–1446; Welicky et al. in press [specimens from Haemulon flavolineatum]

##### Type material.

Holotype (female, TL, W unknown) subocular region of *Haemulon flavolineatum* (USNM 184796); allotype (male, TL, W unknown) (USNM 184797); Paratypes (USNM 184798-184805) (Bunkley Williams and Williams 1981). Not examined.

**Figure 1.**
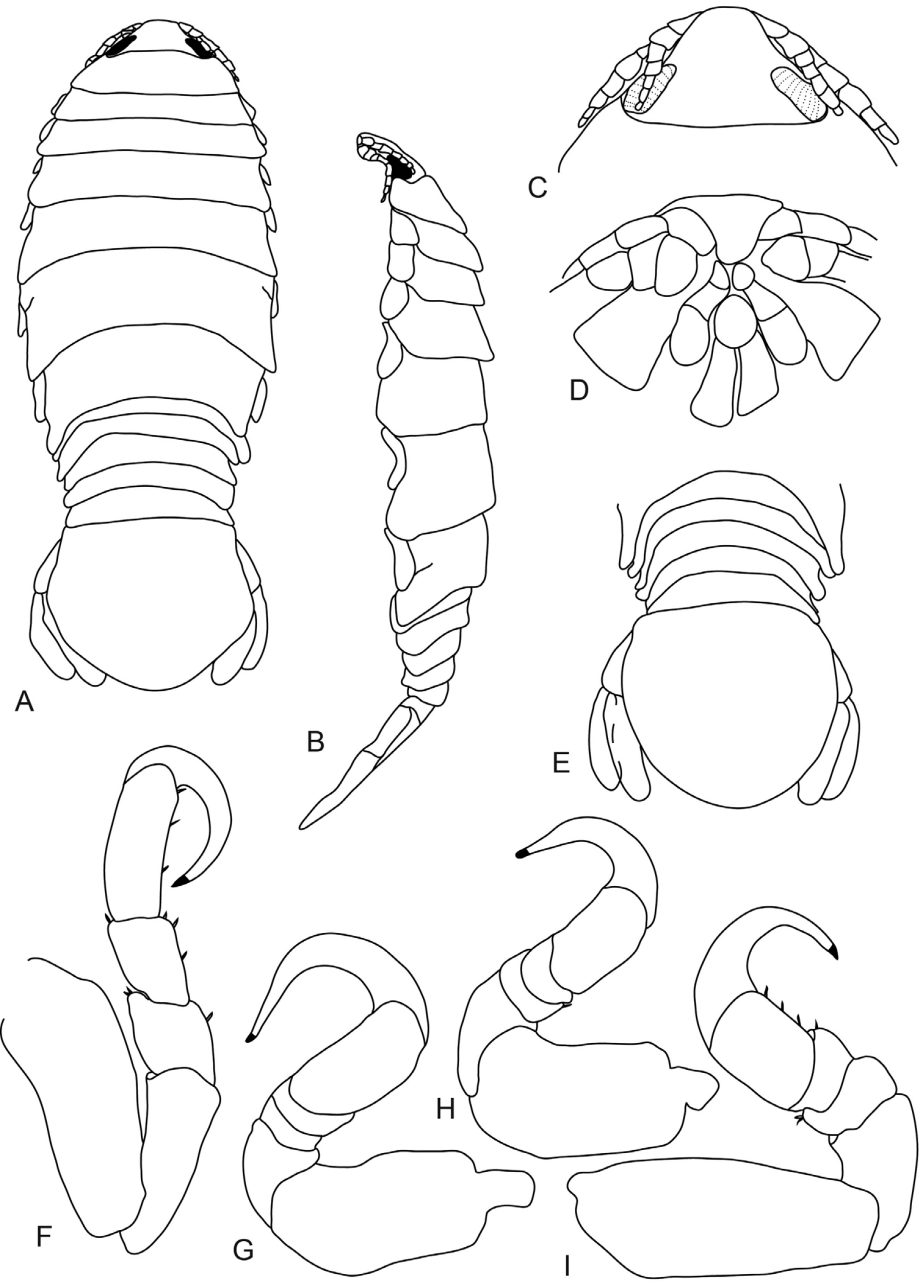
*Anilocra haemuli* female (29 mm) **A–D** *Anilocra haemuli* female (23 mm) **E–I**: **A** dorsal view **B** lateral view **C** dorsal view of cephalon **D** ventral view of cephalon. **E** dorsal pleotelson **F** pereopod 7 **G** pereopod 2 **H** pereopod 1 **I** pereopod 6.

##### Material examined.

All material from the subocular region of *Haemulon flavolineatum*. (TL, W, Voucher Number) Collected by EH and LB Williams: ♀ (32, 13, AMNH_IZC 250203; 32, 14, AMNH_IZC 250204) Mosquito Island, BVI; ♀ (30, 10) West End Enrique Reef, La Parguera, Puerto Rico, 30 Nov 1976; ♀ (30,12, AMNH_IZC 250205) San Cristobal Reef, La Parguera, Puerto Rico, 30 Nov 1976; ♀ (30, 11, AMNH_IZC 250206; 29,14) Mingo Cay, St. John, USVI, 4 Mar 1977; ♀ (32, 13; 34, 14, AMNH_IZC 250207) Lameshur Bay, St. John, USVI, 2 Mar 1977; ♀ (31, 12, AMNH_IZC 250208) West of buoy site, SE of La Parguera, Puerto Rico, 22 Jan 1977. Collected by PC Sikkel and/or ER Brill: ♀ (30, 11; 31, 12) Cinnamon Bay, St. John, Jun 2011; ♀ (28, 12) White Bay, Guana Island, BVI; Jul 2011; ♀ (damaged; 25, 9) St. Thomas, USVI, Jun 2012; ♀ (25, 10) White Bay, Guana Island, BVI, Jul 2012; ♀ (26, 11) Jumbee Bay, St. John, USVI, Jul 2013; ♀ (22, 9; 28, 12) TS (12, 6) White Bay, Guana Island, BVI, Jul-Aug 2016.

**Figure 2.**
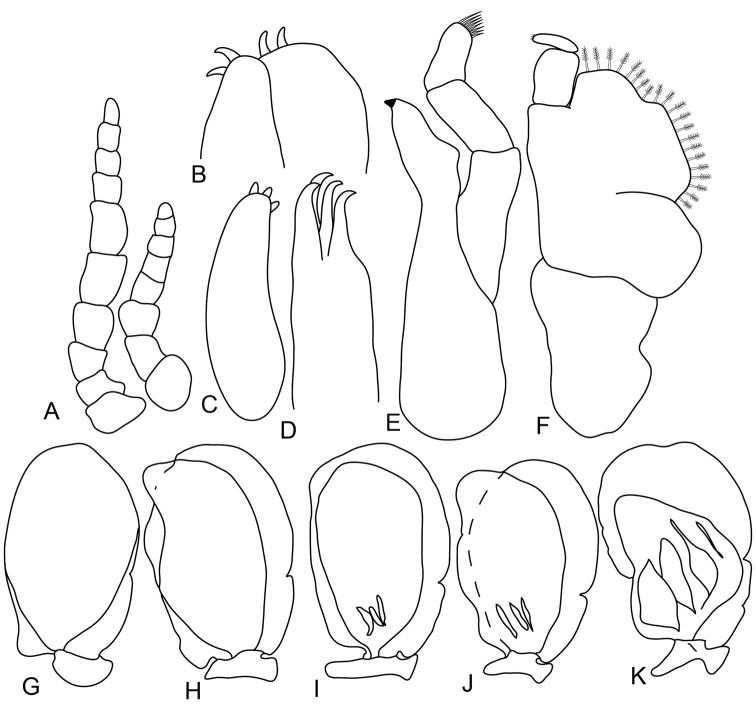
*Anilocra haemuli* female (23 mm) **A, G–K** *Anilocra haemuli* female (25 mm) **B–F**: **A** antenna (left) and antennula (right) **B** maxilla **C** article 3 of maxilliped **D** maxillule **E** mandible **F** maxilliped **G–K** pleopods 1–5 respectively.

##### Ovigerous female.

*Size* intact (29, 13). *Body* weakly ovoid, 2–2.6 times as long as greatest width, dorsal surfaces smooth and polished in appearance, widest at pereonite 5, most narrow at pereonite 1, lateral margins mostly ovate posteriorly. *Cephalon* 0.5–0.7 times longer than wide, visible from dorsal view, weakly trapezoid shaped. *Frontal margin* rounded to form blunt rostrum or simple, not folded. *Eyes* oval with distinct margins, one eye width 0.1–0.2 times width of cephalon; one eye length 0.4–0.5 times length of cephalon. *Pereonite 1* smooth, anterior border straight, anterolateral angle narrowly rounded, not produced. Posterior margins of pereonites smooth and slightly curved laterally. Coxae 2–3 wide; with posteroventral angles rounded; 4–7 rounded and curved; not extending past pereonite posterior margin. Pereonites 1–5 increasing in length and width; 6–7 decreasing in length and width; 1–4 narrower. *Pleon* with pleonite 1 wider than pleonites 2–5, visible in dorsal view; pleonites posterior margin 1–3 posteriorly weakly concave, 4–5 mostly straight. Pleonite 2 not overlapped by pereonite 7; posterolateral angles of pleonite 2 narrowly rounded. Pleonite 1 similar in form to pleonite 2. Pleonite 5 free, not overlapped by lateral margins of pleonite 4, posterior margin straight. Pleotelson 0.9 times as long as anterior width, dorsal surface smooth. *Pleotelson* lateral margins convex, posterior margin narrowly rounded.

**Figure 3.**
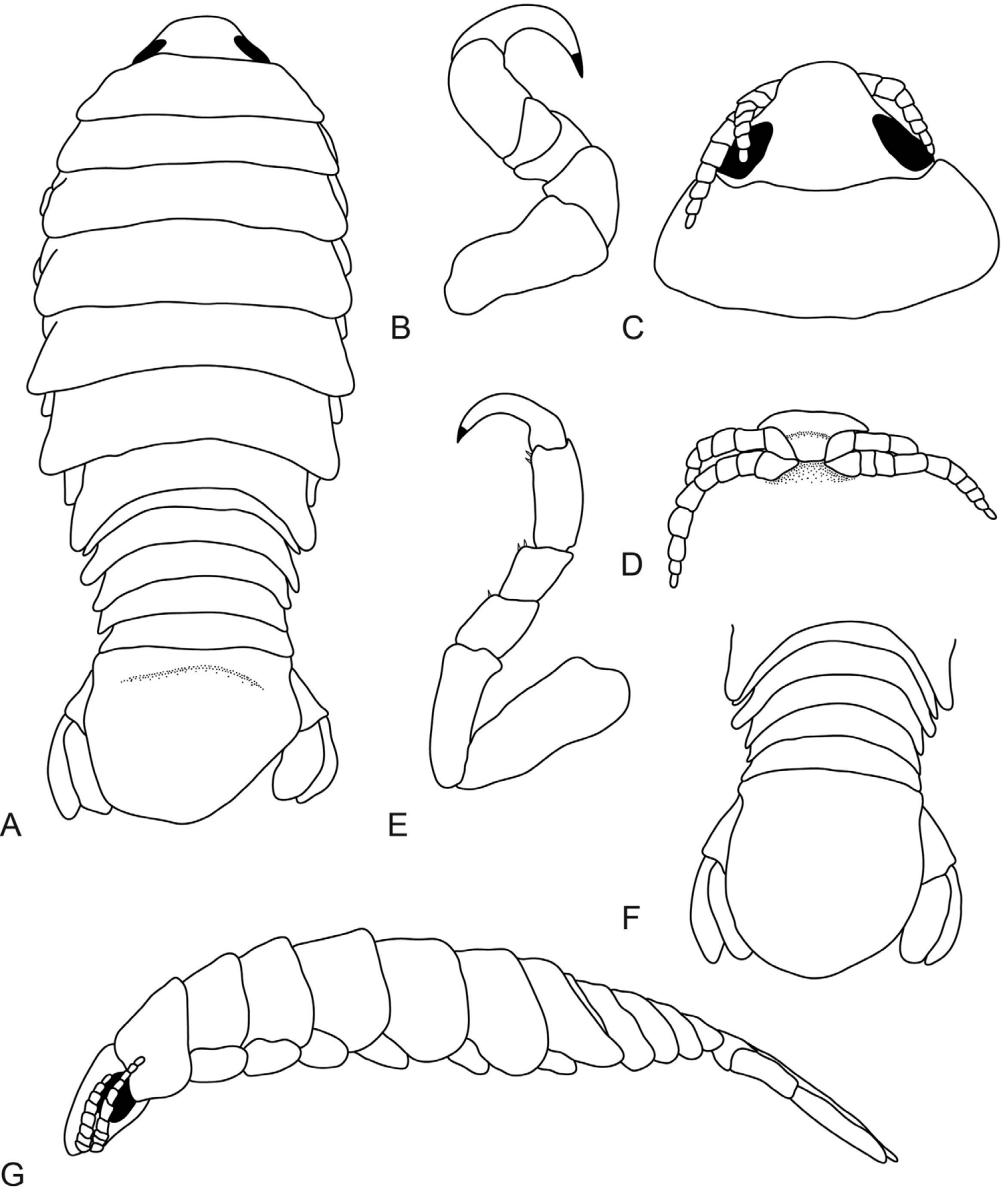
*Anilocra haemuli* transitional stage (12 mm): **A** dorsal view **B** pereopod 1 **C** dorsal view of cephalon **D** ventral view of cephalon **E** pereopod 7 **F** dorsal pleotelson **G** lateral view.

*Antennula* consisting of 7–8 articles; peduncle articles 1 and 2 distinct and articulated; article 2 0.8 times as long as article 1; article 3 0.9 times as long as wide, 0.4 times as long as combined lengths of articles 1 and 2; flagellum with 5 articles, extending to posterior margin of eye. Terminal article with 2 short simple terminal setae. *Antenna* consisting of 10 articles; article 3 1.6 times as long as article 2; article 4 1.2 times as long as wide, 1.5 times as long as article 3; article 5 1.3 times as long as wide, 1.1 times as long as article 4; flagellum with 5 articles, terminal article terminating in 5 short simple setae, extending to middle of pereonite 1. *Mandibular molar process* ending in an acute incisor; mandibular palp article 3 with 7 simple setae. *Maxillula* simple with 4 terminal robust setae. *Maxilla* mesial lobe partly fused to lateral lobe; lateral lobe with 2 recurved robust setae; mesial lobe with 2 recurved robust setae. *Maxilliped* weakly segmented, with lamellar oostegite lobe, article 3 with 3 small robust setae.

*Pereopod 1* basis 1.7 times as long as greatest width; ischium 0.7 times as long as basis; merus proximal margin without bulbous protrusion; carpus with straight proximal margin; propodus 1.3 times as long as wide; dactylus stout, 2.7 times as long as propodus, 3.8 times as long as wide. *Pereopod 2* propodus 2.1 times as long as wide; dactylus 2.2 as long as propodus. *Pereopod 6* basis 2.6 times as long as greatest width; ischium 0.5 times as long as basis; propodus 1.3 times as long as wide; dactylus 2.5 times as long as propodus. *Pereopod 7* basis 3.2 times as long as greatest width; ischium 0.7 times as long as basis, without protrusions; merus proximal margin without bulbous protrusion; merus 1.1 times as long as wide, 1.6 times as long as ischium; carpus 1.5 times as long as wide, 0.5 times as long as ischium, without bulbous protrusion; propodus 2.6 times as long as wide, 0.8 times as long as ischium; dactylus slender, 1.8 times as long as propodus, 5.0 times as long as wide. Pereopod 7 with few setae on propodus, carpus, and merus.

**Figure 4.**
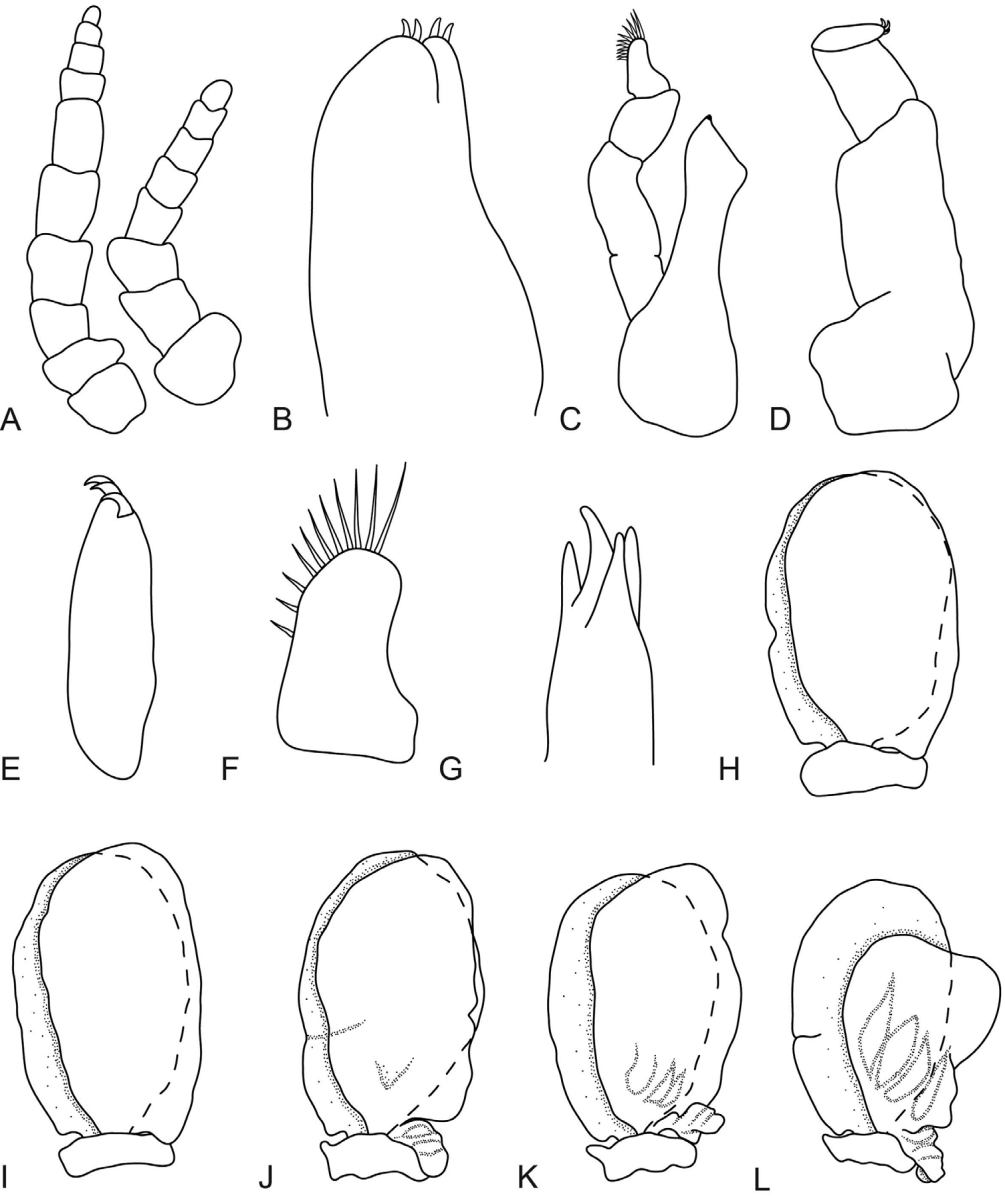
*Anilocra haemuli* transitional stage (12 mm): **A** antenna (left) and antennula (right) **B** maxilla **C** mandible **D** maxilliped **E** article 3 of maxilliped **F** article 3 of mandibular palp **G** maxillule **H–K** pleopods 1–5 respectively.

*Pleopods* without setae, exopod larger than endopod. Pleopod 1 exopod 1.5 times as long as wide, lateral margin weakly convex, distally narrowly rounded, medial margin weakly oblique, mesial margin weakly convex; endopod 1.6 times as long as wide, lateral margin weakly convex, distally narrowly rounded, mesial margin slightly convex; peduncle twice as wide as long, without retinaculae, pointed projection on lateral margin. Pleopods 2–5 similar to pleopod 1. Pleopods 3–5 endopods proximal borders do not extend below exopod to peduncle, fleshy lobes and medial lobes present. Peduncle lobes absent.

*Uropod* length equal length of pleotelson; peduncle 0.7–0.9 times longer than rami, lateral margin without setae; rami not extending beyond pleotelson, marginal setae absent, apices broadly rounded. *Endopod* apically rounded, 3.1–3.5 times as long as greatest width. *Exopod* not extending to end of endopod, 3.8–4.4 times as long as greatest width, apically rounded, lateral margin weakly convex, mesial margin weakly convex, terminating without setae.

##### Transitional stage.

*Size* (12, 6). Similar to female but smaller. *Body* 2.5 times as long as wide. *Antennula* bases separated, consisting of 8 articles, extending to posterior margin of eye. *Antenna* consisting of 10 articles, extending to middle of pereonite 1. *Mandibular molar process* ending in an acute incisor; mandibular palp article 3 with 11 simple setae. *Maxillula* simple with 4 terminal robust setae. *Maxilla* mesial lobe partly fused to lateral lobe; lateral lobe with 2 recurved robust setae; mesial lobe with 2 recurved robust setae. *Maxilliped* weakly segmented, with lamellar oostegite lobe, article 3 with 3 small recurved robust setae. *Pereopod 7* with few small robust setae on carpus, merus and propodus. *Pleopod 2* appendix masculina absent.

##### Distribution.

Off the coast of southern Florida (USA) and throughout the Caribbean (Bunkley Williams and Williams 1981; Welicky et al. 2013, Welicky and Sikkel 2014, 2015, Welicky et al. in press).

##### Hosts.

Known from *Haemulon flavolineatum* (Desmarest, 1823), *H. aurolineatum* (Cuvier, 1830), *H. carbonarium* (Poey, 1860), *H. chrysargyreum* (Günther, 1859), *H. macrostomum* (Günther, 1859) *H. plumieri* (Lacépède, 1801), *H. sciurus* (Shaw, 1803). Host records previously reported and which should be verified in the future: *Cephalopholis cruentaus* (Lacepède, 1802; formerly reported and classified as *Epinephelus cruentatus*, Lacepède, 1802) , *C. fulva* (Linnaeus, 1758; formerly reported and classified as *Epinephelus fulvus* Linnaeus, 1758), *Epinephelus guttatus* (Linnaeus, 1758), *Paranthias furcifer* (Valenciennes, 1828), *Mycteroperca rubra* (Bloch, 1793), *M. bonaci* (Poey, 1860), and *Orthopristis ruber* (Cuvier, 1830).

##### Remarks.

The description of *A. haemuli* from *H. flavolineatum* given above is in agreement with the original description in Bunkley Williams and Williams (1981). We supplement the original species diagnosis by now providing drawings and measurements of the antenna and antennula articles, additional pereopods, and pleopods.

*Anilocra haemuli* from *H. flavolineatum* can be distinguished from all other Caribbean species based on the morphological and/or site attachment differences among species that were reported in Bunkley Williams and Williams (1981). Pereopods 2–4 do not swell on the outer margin of the dactyl, thereby excluding it from being *Anilocra adudefdufi* Bunkley Williams & Williams, 1981, *A. holocanthi* Bunkley Williams & Williams, 1981, *A. chaetodontis*, or *A. partiti* Bunkley Williams & Williams, 1981. In *A. haemuli*, the posterioventral angle of pereonite 6 is slightly produced thereby excluding it from being *A. holocentri*. The The endopod of the uropod of *A. haemuli* extends beyond the posterior end of the exopod, which is not the case in *Anilocra chromis* or *A. partiti*. Whereas the attachment site of *A. haemuli* is under the eye, *A. holocentri* and *A. myripristis* Bunkley Williams & Williams, 1981 attach between the eyes, and *A. acanthuri* Bunkley Williams & Williams, 1981 attaches under the pectoral fin.

#### Anilocra brillae sp. n.

Taxon classificationAnimaliaIsopodaCymothoidae

http://zoobank.org/0D6D3D87-D9AD-46E3-B976-9A77D7245E34

Figs 5 , 6 , 7 , 8

Anilocra haemuli (Part) of Bunkley Williams and Williams (1981) [records from Serranidae].

##### Material examined.

All material from the subocular region of *Epinephelus guttatus*.

Holotype. Ovigerous ♀ (38, 17, AMNH_IZC 250209), Lameshur Bay, St. John, 18°18'59"N, 64°43'25"W, US Virgin Islands, 2 Mar 1977, coll. EH and LB Williams.

Paratype. Ovigerous ♀ dissected (39, 15, AMNH_IZC 250210), Lameshur Bay, St. John, USVI, 2 Mar 1977 by EH and LB Williams.

##### Others examined.

Collected by EH and LB Williams: ♀ (33, 13, AMNH_IZC 250211; 24, 9) San Cristobal Reef, La Parguera, Puerto Rico 28–29 Jan 1977; ♀ (35, 15, AMNH_IZC 250212; 32, 13 AMNH_IZC 250213) Lameshur Bay, St. John, USVI, 2 Mar 1977; ♀ (39, 16, AMNH_IZC_250214) Buck Island, St. Thomas, USVI, 5 Mar 1977; ♀ (34, 15, AMNH_IZC 250215; 25, 10 AMNH_IZC 25016) Laurel Reef, La Parguera, Puerto Rico, 18 May 1977; ♀ (30, 12) Ensenada Honda, Vieques, Puerto Rico, 20 Dec 1983. Collected by PC Sikkel and ER Brill: ♀ (27, 10; 30, 13; 26, 10; 31, 12; 29, 12; 29, 12; damaged) TS (11,6) White Bay, Guana Island, 18°28'0"N, 64°33'59"W, BVI, Jul-Aug 2016.

**Figure 5.**
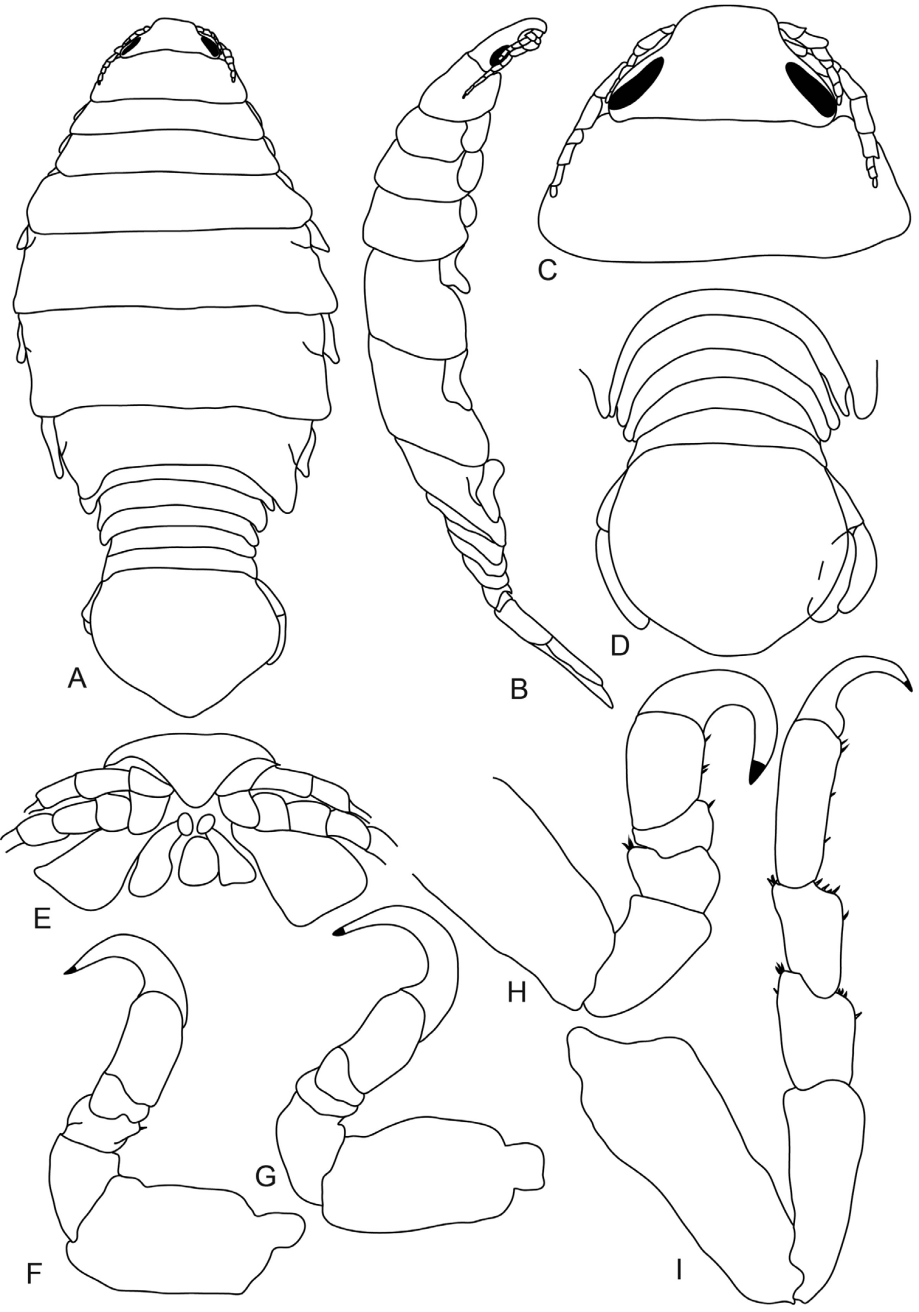
*Anilocra brillae* sp. n. female holotype (38 mm)(AMNH_IZC 250209) **A–E** *Anilocra brillae* sp. n. female paratype (39 mm) (AMNH_IZC 250210) **F–I**: **A** dorsal view **B** lateral view **C** dorsal view of cephalon **D** pleotelson **E** ventral view of cephalon **F** pereopod 1 **G** pereopod 2 **H** pereopod 6 **I** pereopod 7.

**Ovigerous female.** *Size* (38, 17). *Body* ovoid, 2.1–2.4 times as long as greatest width, dorsal surfaces smooth and polished in appearance, widest at pereonite 5, most narrow at pereonite 1, lateral margins mostly posteriorly ovate. *Cephalon* 0.5–0.7 times longer than wide, visible from dorsal view, trapezoid shaped. *Frontal margin* rounded to form blunt rostrum, not folded. *Eyes* oval with distinct margins, one eye width 0.1 times width of cephalon; one eye length 0.5–0.6 times length of cephalon. *Pereonite* 1 smooth, anterior border straight, anterolateral angle narrowly rounded, not produced. Posterior margins of pereonites smooth and slightly curved laterally. Coxae 2–3 wide with posteroventral angles rounded; 4–7 with narrowly produced point, curved; not extending past pereonite posterior margin. Pereonites 1–5 increasing in length and width; 6–7 decreasing in length and width; 5 and 6 subequal in width, 1–4 narrower. *Pleon* with pleonite 1 most wide, visible in dorsal view; pleonites posterior margin smooth, 1–4 posteriorly concave, 5 straight. Pleonite 2 not overlapped by pereonite 7; posterolateral angles of pleonite 2 narrowly rounded. Pleonite 1 differ in form to pleonite 4 and 5, similar to pleonite 2 and 3. Pleonite 5 equal width to pleonite 4, not overlapped by lateral margins of pleonite 4, posterolateral angles narrowly rounded, posterior margin straight. *Pleotelson* 1.1–1.4 times as long as anterior width, dorsal surface smooth, lateral margins convex, posterior margin converging to weak caudomedial point.

*Antennula* bases separated, shorter than antenna, consisting of 7–9 articles; peduncle articles 1 and 2 distinct and articulated; article 2 1.5 times as long as article 1; article 3 0.9 times as long as wide, 0.5 times as long as combined lengths of articles 1 and 2; flagellum with 4 articles, extending to posterior margin of eye. Terminal article terminating in 1 short simple seta. *Antenna* comprised of 9–10 articles, peduncle article 3 1.5 times as long as article 2; article 4 1.3 times as long as wide, 1.1 times as long as article 3; article 5 1.6 times as long as wide, 1.1 times as long as article 4; flagellum with 4 articles, terminal article with 5 short simple setae, extending to posterior of pereonite 1. *Mandibular molar process* ending in an acute incisor; mandibular palp article 3 with 8 simple setae. *Maxillula* simple with 4 terminal robust setae. *Maxilla* mesial lobe partly fused to lateral lobe; lateral lobe with 2 recurved robust setae; mesial lobe with 1 recurved robust seta. *Maxilliped* weakly segmented, with lamellar oostegite lobe, article 3 with 3 recurved robust setae.

**Figure 6.**
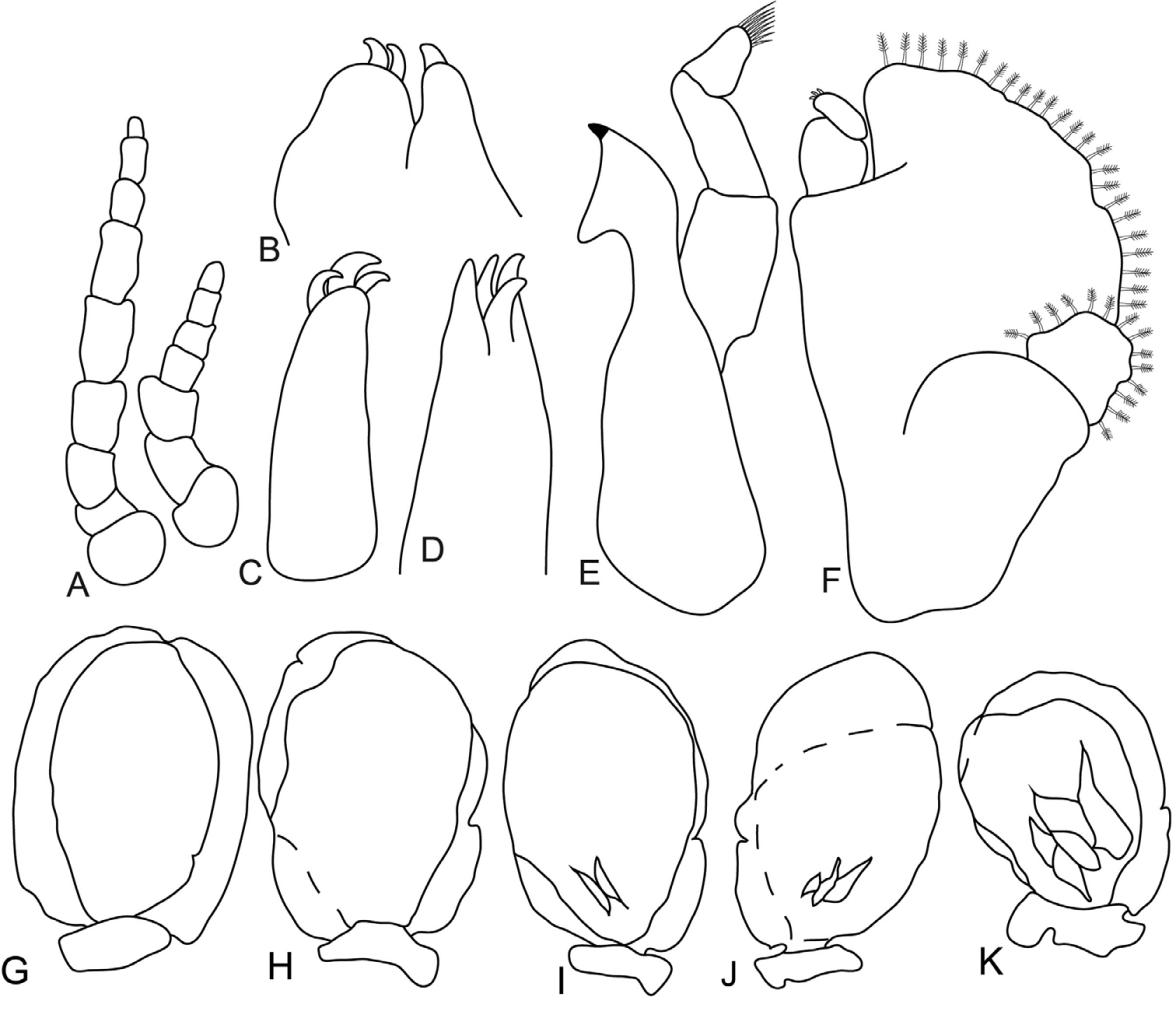
*Anilocra brillae* sp. n. female paratype (39 mm) (AMNH_IZC 250210) **A, G–K** *Anilocra brillae* sp. n. female (pleotelson damaged) **B–F**: **A** antenna (left) and antennula (right) **B** maxilla **C** article 3 of maxilliped **D** maxillule **E** mandible **F** maxilliped **G–K** pleopods 1–5 respectively.

*Pereopod 1* basis 1.8 times as long as greatest width; ischium 0.23 times as long as basis; merus proximal margin without bulbous protrusion; carpus with straight proximal margin; propodus 1.9 times as long as wide; dactylus moderately slender, 1.8 times as long as propodus, 3.7 times as long as wide. *Pereopod 2* propodus 1.7 as long as wide; dactylus 2.7 times as long as propodus, 4.9 times as long as wide. Pereopods gradually increasing in size towards posterior. *Pereopod 6* basis 1.7 times as long as greatest width; ischium 0.7 times as long as basis; propodus 1.5 times as long as wide, dactylus 2.3 times as long as propodus, 3.8 times as long as wide. *Pereopod 7* basis 3.0 times as long as greatest width; ischium 0.7 times as long as basis, without protrusions; merus proximal margin without bulbous protrusion, 2.0 times as long as wide, 0.7 times as long as ischium; carpus 1.5 times as long as wide, 0.6 times as long as ischium, without bulbous protrusion; propodus 3.2 times as long as wide, 0.8 times as long as ischium; dactylus moderately slender, 0.9 times as long as propodus, 3.5 times as long as wide. Pereopod 7 with many setae on propodus, carpus, and merus.

*Pleopods* without setae, exopod larger than endopod. Pleopod 1 exopod 1.2 times as long as wide, lateral margin weakly convex, distally narrowly rounded, medial margin weakly oblique, mesial margin weakly convex; endopod 1.8 times as long as wide, lateral margin weakly convex, distally narrowly rounded, mesial margin slightly convex, peduncle 2.2 times as wide as long, with pointed projection on lateral margin. Pleopods 2–5 similar to pleopod 1. Pleopods 3–5 endopods proximal borders do not extend below exopod to peduncle, fleshy lobes and medial lobes present. Peduncle lobes absent.

**Figure 7.**
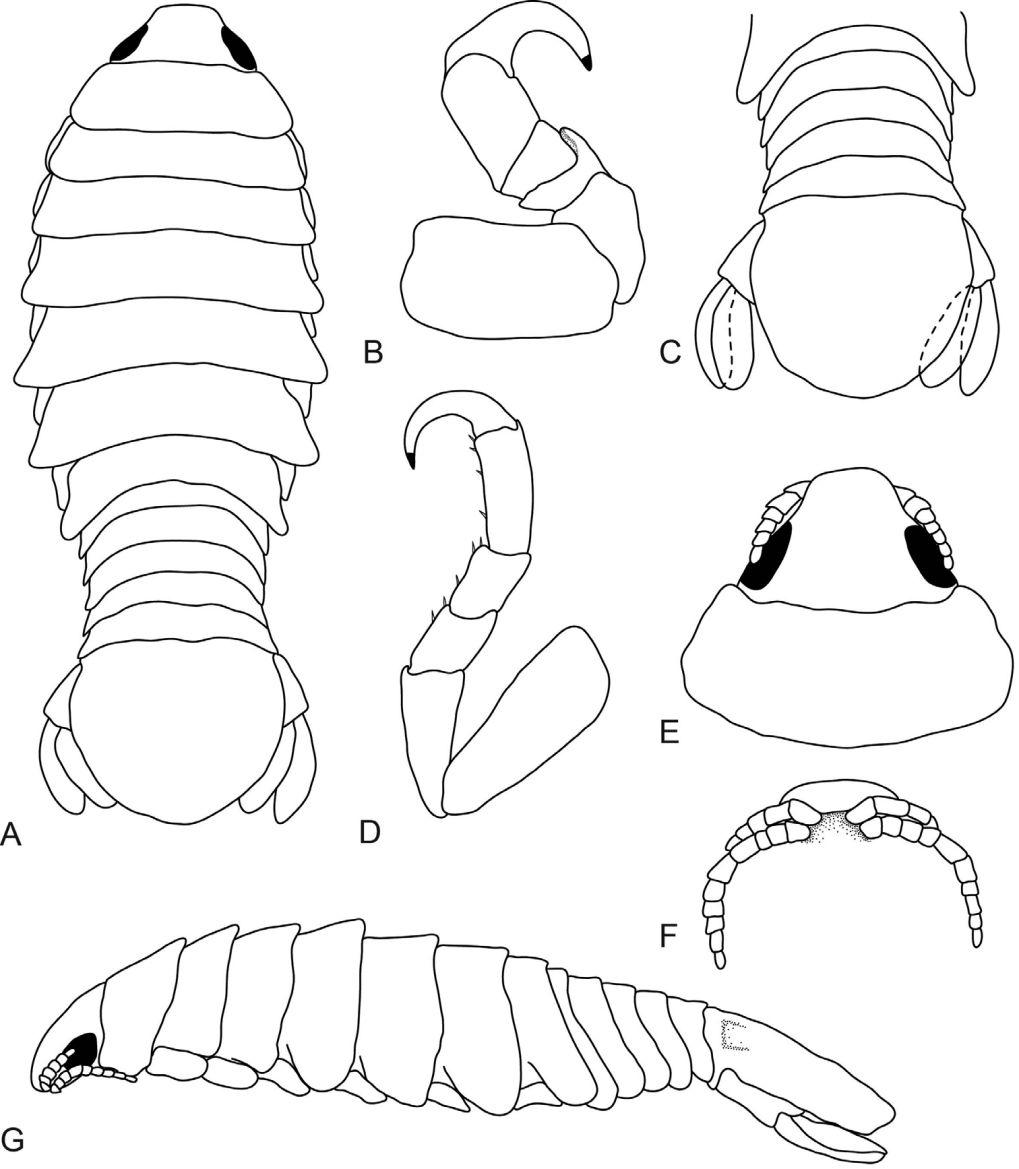
*Anilocra brillae* sp. n. transitional stage (11 mm): **A** dorsal view **B** pereopod 1 **C** dorsal pleotelson **D** pereopod 7 **E** dorsal view of cephalon **F** ventral view of cephalon **G** lateral view.

*Uropod* more than half the length of pleotelson, peduncle 0.7 times longer than rami, peduncle lateral margin without setae; rami not extending beyond pleotelson, marginal setae absent, apices broadly rounded. *Endopod* apically rounded, 2.2 times as long as greatest width, lateral margin weakly convex, mesial margin weakly convex, terminating without setae. *Exopod* not extending to end of endopod, 2.6 times as long as greatest width, apically rounded, lateral margin convex, mesial margin weakly convex or weakly concave, terminating without setae.

##### Transitional stage.

*Size* (11, 6). Similar to female but smaller. *Body* 2.6 times as long as wide. *Antennula* bases separated, consisting of 8 articles, extending to middle of eye. *Antenna* consisting of 10 articles, extending to middle of pereonite 1. *Mandibular molar process* ending in an acute incisor; *mandibular palp* article 2 with 2 simple setae, article 3 with 7 simple setae. *Maxillula* simple with 4 terminal robust setae. *Maxilla* mesial lobe partly fused to lateral lobe; lateral lobe with 2 recurved robust setae; mesial lobe with 2 recurved robust setae. *Maxilliped* weakly segmented, with lamellar oostegite lobe, article 3 with 3 recurved robust setae. *Pereopod 7* with several small robust setae on carpus, merus and propodus. *Pleopod 2* appendix masculina absent.

##### Etymology.

This species is named in honor of Elizabeth R. Brill for her dedication to studying the ecology of *A. haemuli*, and for collecting many of the *A. haemuli* and *A. brillae* sp. n. specimens used in this study.

##### Distribution.

Known from St. John and St. Thomas, USVI, Guana Island, BVI, and islands of Puerto Rico, Spanish Virgin Islands. Expected distribution throughout the Caribbean Sea, where fish of the Serranidae family inhabit.

##### Hosts.

Known from *Epinephelus guttatus* (Linnaeus, 1758).

##### Remarks.

Previously, *A. brillae* sp. n. was identified as *A. haemuli*. Compared to *A. haemuli*, the outer margins of the cephalon and pereonites 1–4 of *A. brillae* sp. n. form a more pronounced trapezoid shape and the remaining portion of the body is ovoid. *A. brillae* sp. n has more strongly narrowed pleonites than *A. haemuli*. Pleonites 1–3 of *A. brillae* sp. n. are wider than 4–5 and 4–5 are subequal, whereas the pleonites 1–2 of *A. haemuli* are wider than 3–5, and 3–5 are subequal. Pleonite 5 is more posteriorly rounded in *A. brillae* sp. n, but this is somewhat variable among individuals. Another more variable feature is *A. brillae* sp. n. has a more caudomedially pointed pleotelson than *A. haemuli*. Typically, the seventh pereopod of *A. brillae* sp. n. is proportionally larger, has more robust setae, and the setae are distributed more extensively over the articles when compared to *A. haemuli*. The antennula peduncle of *A. brillae* sp. n. is regularly observed as shorter and more robust than that of *A. haemuli*. With respect to attachment, both species infest the subocular region, and if infested by two parasites, one parasite typically attaches under each eye. Infestation by a third *A. brillae* sp. n. on a single host seems to occur with more frequency than tertiary infestation by *A. haemuli* on a single host. The third parasite is typically attached between the eyes on the head of the host, or adjacent to one of the other parasites (RLW, pers obs).

**Figure 8.**
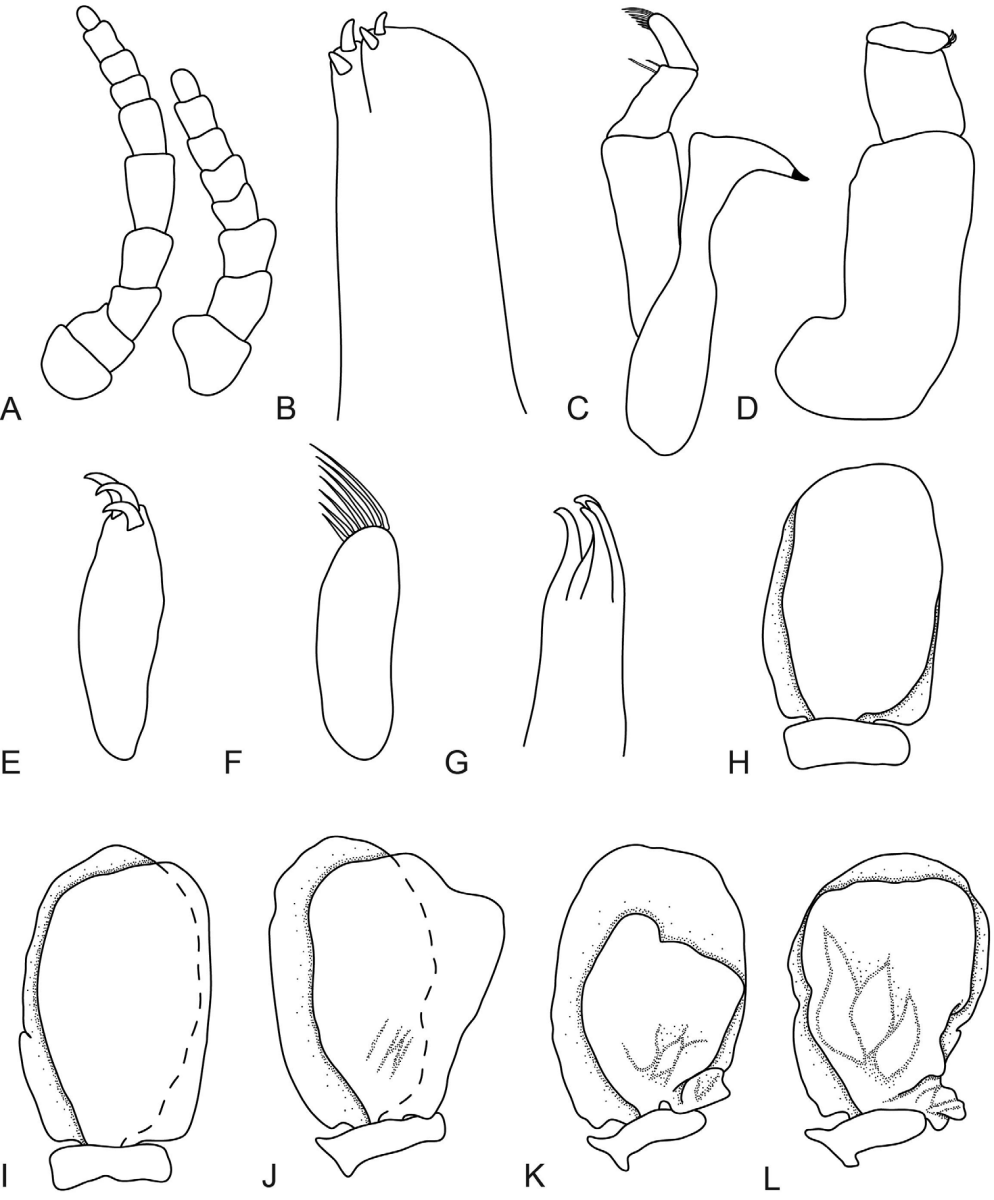
*Anilocra brillae* sp. n. transitional stage (11 mm): **A** antenna (left) and antennula (right) **B** maxilla **C** mandible **D** maxilliped **E** article 3 of maxilliped **F** article 3 of mandibular palp **G** maxillule **H–K** pleopods 1–5 respectively.

*Anilocra brillae* sp. n. can be distinguished from all other Caribbean species except *Anilocra haemuli* using the same morphological comparisons described between *A. haemuli* and other *Anilocra* spp. given in Bunkley Williams and Williams (1981). Additionally, the body of *A. brillae* sp. n. is not expanded and is more elongate compared to the bodies of *A. holocanthi* and *A. chaetodontis*.

## Discussion

The results of this study provide the first reliable COI sequences for species of *Anilocra*, and confirm that *A. haemuli* from *H. flavolineatum* is morphologically and genetically different than the *Anilocra* specimens collected from *E. guttatus*, and are here described as *A. brillae* sp. n. Our morphological data suggest there are two different species given the number of differences consistently observed, and our molecular analyses demonstrate a 4% difference between *A. haemuli* and *A. brillae* sp. n. This difference is less than half of that observed between *A. brillae* sp. n. and *A. chromis*, which are more conspicuously morphologically different. Our supplemental analyses were conducted utilizing the available *Anilocra* sp. COI sequences on GenBank, and there was a high level of interspecific divergence of these sequences compared with our dataset. The large differences in interspecific divergence between the specimens of this study and those provided on GenBank may be explained by the fact that the GenBank specimens may have been misidentified or not identified at all, as no morphological identification was described in Ketmaier et al. (2007). Thus, further interspecific comparisons cannot be assessed at this time.

*Anilocra* spp. have been reported to influence the fitness (Adlard and Lester 2004, Fogelman et al. 2009) and behavior (Meadows and Meadows 2003, Welicky and Sikkel 2015, Welicky et al. in press) of their fish hosts, and *Anilocra brillae* sp. n. infests *E. guttatus*, a grouper species that is currently recovering from previously intense fishing pressure (Nemeth et al. 2005). There is limited knowledge on the biotic stressors that influence *E. guttatus* population dynamics, and thus the effects of *A. brillae* sp. n. on *E. guttatus* should be examined as a potential stressor. Moreover, by studying this host-parasite interaction, further insight into variations in life histories of *Anilocra* spp. may be gained, if the life cycle of the parasite coincides with that of their host. The only complete description of an *Anilocra* spp. life cycle is of a species that infests an egg laying/guarding fish species (Adlard and Lester 1995), whereas many *Anilocra* spp. infest broadcast spawners. Interestingly, *A. brillae* sp. n. infests a fish species that undergoes an annual long distance migration to spawn in an aggregation (Nemeth 2011). Given that *Anilocra* spp. infection has been reported to influence host swimming performance in some fish (e.g., Binning et al. 2013), *A. brillae* sp. n. infection may indirectly influence the reproductive success of their hosts.

This study exemplifies that there is an incomplete but growing knowledge of cymothoid life histories, genetics, and morphology, and how these disciplines relate to host-parasite ecology. Continued efforts to conduct studies in these disciplines are necessary to better understand one of the least understood parasite families.

## Supplementary Material

XML Treatment for Anilocra

XML Treatment for Anilocra haemuli

XML Treatment for Anilocra brillae

## Supplementary materials

Supplementary material 1 Basepair differences of *Anilocra* spp. Data type: statistical data Explanation note: **The number identifier in the horizontal header column represents the number and corresponding species listed in the vertical column header.** This dataset is made available under the Open Database License (http://opendatacommons.org/licenses/odbl/1.0/). The Open Database License (ODbL) is a license agreement intended to allow users to freely share, modify, and use this Dataset while maintaining this same freedom for others, provided that the original source and author(s) are credited.Rachel L. Welicky, Kerry A. Hadfield, Paul C. Sikkel, Nico J. Smit

Supplementary material 2 Kimura 2-Parameter (K2P) distance of *Anilocra* spp. Data type: statistical data Explanation note: K2P distance expressed in percent. The number identifier in the horizontal header column represents the number and corresponding species listed in the vertical column header. This dataset is made available under the Open Database License (http://opendatacommons.org/licenses/odbl/1.0/). The Open Database License (ODbL) is a license agreement intended to allow users to freely share, modify, and use this Dataset while maintaining this same freedom for others, provided that the original source and author(s) are credited.Rachel L. Welicky, Kerry A. Hadfield, Paul C. Sikkel, Nico J. Smit
